# Network Pharmacology, Molecular Dynamics and In Vitro Assessments of Indigenous Herbal Formulations for Alzheimer’s Therapy

**DOI:** 10.3390/life14101222

**Published:** 2024-09-25

**Authors:** Oluwafemi Adeleke Ojo, Omolola Adenike Ajayi-Odoko, Gideon Ampoma Gyebi, Damilare IyinKristi Ayokunle, Akingbolabo Daniel Ogunlakin, Emmanuel Henry Ezenabor, Adesoji Alani Olanrewaju, Oluwatobi Deborah Agbeye, Emmanuel Tope Ogunwale, Damilare Emmanuel Rotimi, Dalia Fouad, Gaber El-Saber Batiha, Oluyomi Stephen Adeyemi

**Affiliations:** 1 Good Health and Wellbeing Research Clusters (SDG 03), Bowen University, Iwo 232102, Nigeria; gbolaogunlakin@gmail.com (A.D.O.); emmanuel.ezenabor@bowen.edu.ng (E.H.E.); oluwatobiagbeyed@gmail.com (O.D.A.); oluyomiadeyemi@gmail.com (O.S.A.); 2 Phytomedicine, Molecular Toxicology, and Computational Biochemistry Research Group, Biochemistry Programme, Bowen University, Iwo 232102, Nigeria; 3 Microbiology Programme, Bowen University, Iwo 232102, Nigeria; omolola.ajayi@bowen.edu.ng; 4 Natural Products and Structural (Bio-Chem)-Informatics Research Laboratory (NpsBC-RI), Department of Biochemistry, Bingham University, Karu 961105, Nigeria; gideonagyebi@gmail.com; 5 Department of Biotechnology and Food Science, Faculty of Applied Sciences, Durban University of Technology, P.O. Box 1334, Durban 4000, South Africa; 6 Pure and Applied Biology Programme, Bowen University, Iwo 232102, Nigeria; opeoluwa02@gmail.com; 7 Chemistry and Industrial Chemistry Programme, Bowen University, Iwo 232102, Nigeria; adesoji.olanrewaju@bowen.edu.ng; 8 Animal Science Programme, Bowen University, Iwo 232102, Nigeria; tope.ogunwale@bowen.edu.ng; 9 Department of Pharmacology and Pharmaceutical Sciences, Alfred E. Mann School of Pharmacy and Pharmaceutical Sciences, University of Southern California, Los Angeles, CA 90089, USA; rotimidamilare1@gmail.com; 10 Department of Biochemistry, Landmark University, Omu-Aran 251101, Nigeria; 11 Department of Zoology, College of Science, King Saud University, P.O. Box 22452, Riyadh 11495, Saudi Arabia; dibrahim@ksu.edu.sa; 12 Department of Pharmacology and Therapeutic, Faculty of Veterinary Medicine, Damanhour University, Damanhour 22511, AlBeheira, Egypt; gaberbatiha@gmail.com; 13 Laboratory of Sustainable Animal Environment Systems, Graduate School of Agricultural Sciences, Tohoku University, Sendai 980-8579, Japan

**Keywords:** medicinal plants, network pharmacology, experimental analyses, neurodegenerative disorders

## Abstract

Alzheimer’s disease (AD) is an age-associated neurodegenerative condition marked by amyloid plaques, synaptic dysfunction, and neuronal loss. Besides conventional medical care, herbal therapies, both raw and refined, have attracted researchers for their potential therapeutic effects. As a proof-of-concept, our study combined HPLC-DAD analysis of bioactive constituents, network pharmacology, molecular dynamics (MD), molecular docking, post-MD analysis, and experimental verification to investigate the mechanisms of crude drug formulations as a therapeutic strategy for AD. We identified nine bioactive compounds targeting 188 proteins and 1171 AD-associated genes. Using a Venn diagram, we found 47 overlapping targets, forming “herb-compound-target (HCT)” interaction networks and a protein‒protein interaction (PPI) network. Simulations analyzed binding interactions among the three core targets and their compounds. MD assessed the stability of the best-ranked poses and beneficial compounds for each protein. Among the top 22 hub genes, AChE, BChE, and MAO, ranked 10, 14, and 34, respectively, were selected for further analysis. Two tetraherbal formulations, Form A and Form B, showed notable activity against AChE. Form A exhibited significant (*p* < 0.0001) inhibitory activity (IC50 = 114.842 ± 2.084 µg/mL) compared to Form B (IC50 = 142.829 ± 4.258 µg/mL), though weaker than galantamine (IC50 = 27.950 ± 0.122 µg/mL). Form B had significant inhibitory effects on BChE (IC50 = 655.860 ± 32.812 µg/mL) compared to Form A (IC50 = 679.718 ± 20.656 µg/mL), but lower than galantamine (IC50 = 23.126 ± 0.683 µg/mL). Both forms protected against Fe2+-mediated brain injury by inhibiting MAO. Docking identified quercetin (−10.2 kcal/mol) and myricetin (−10.1 kcal/mol) for AChE; rutin (−10.6 kcal/mol) and quercetin (−9.7 kcal/mol) for BChE; and kaempferol (−9.1 kcal/mol) and quercetin (−8.9 kcal/mol) for MAO. These compounds were thermodynamically stable based on MD analysis. Collectively, the results offer a scientific rationale for the use of these specifically selected medicinal herbs as AD medications.

## 1. Introduction

Alzheimer’s disease (AD) is an age-associated, progressive neurodegenerative condition characterized by amyloid plaques, neuronal cell death, synaptic disorders, and neurofibrillary tangles. One of the main factors contributing to AD pathogenesis is the accumulation of β-amyloid, which detrimentally affects synaptic activity, leading to neurodegeneration [1]. This condition predominantly affects elderly people, representing a significant global health concern, with dementia impacting 47 million people in 2015, a number projected to increase to 131 million by 2050. The risk of developing Alzheimer’s disease is significantly influenced by dietary practices, as various studies have highlighted the potential preventive effects of bioactive compounds from diverse food sources to potentiate drug development [2].

Current AD treatments mainly include N-methyl-D-aspartate (NMDA) receptor inhibitors and acetylcholinesterase inhibitors (AChE). However, these medicines often target a single pathway, exhibit low resistance to drug effects, and can cause adverse side effects, resulting in suboptimal clinical outcomes [3]. In light of the challenges associated with conventional treatments, there has been a growing interest in traditional herbal remedies as alternatives that address the limitations of synthetic drugs [4].

Herbal medicine offers several promising strategies for slowing AD progression and managing symptoms, primarily through mechanisms such as reducing oxidative stress, modulating inflammatory pathways, inhibiting acetylcholinesterase activity, and promoting neuroprotection. For instance, the antioxidative properties of certain herbs can mitigate the oxidative stress implicated in AD pathogenesis, while others may exert anti-inflammatory effects by downregulating pro-inflammatory cytokines. The production and sale of plant-derived medications are increasing, underscoring their increasing scientific and financial importance in healthcare [5].

This research investigated three beneficial herbal plants, *Beta vulgaris* (root and leaves), *Persea americana* (avocado) seeds, and *Syzygium aromaticum* (cloves), to identify novel treatment strategies for AD. Beta vulgaris is a member of the Chenopodiaceae family and is valued for its medicinal qualities and juice value [6]. It contains biologically active components such as flavonoids, ascorbic acid, saponins, nitrate, and polyphenols that contribute to its health benefits, including antioxidant, anti-inflammatory, and antihypertensive effects [7,8].

*Persea americana*, or avocado, belongs to the Lauraceae family and is well known worldwide because of its nutritionally rich fruit [9]. Seeds contain phytocompounds, including flavonoids, glycosides, alkaloids, phenols, and tannins, which have various medicinal uses, including for the treatment of diarrhea, dysentery, and hypertension [10].

*Syzygium aromaticum*, also referred to as clove, is a member of the Myrtaceae family and is indigenous to parts of India and Indonesia [11]. Cloves are recognized for their anti-inflammatory, antimicrobial, and antioxidant qualities, attributed to their bioactive constituents, including vitamin A and beta-carotene [12,13].

To advance drug discovery, the concept of network pharmacology has been introduced. This approach has been increasingly used over the past decade to understand the mechanisms of herbal formulas by examining the complex interactions between ligands, targets, and biological systems [14]. Molecular docking, a computational approach, is also used to determine the optimal binding configuration of a ligand and a molecular target. Network pharmacology aims to methodically and systematically examine the regulatory impacts of drugs on biomolecular networks, thereby advancing experimental studies conducted in vivo, ex vivo, and in vitro [15]. This study investigated the effects of distinct plant formulations combined with active bioactive compounds in combating AD through the use of a combination of network pharmacology and molecular dynamics modeling.

## 2. Methods

### 2.1. Plant Materials

The selected medicinal plants, namely, *Beta vulgaris* root and leaves, *Persea americana* seeds, and *Syzygium aromaticum*, were collected from different areas in Nigeria. Specifically, the plants were collected from the following locations: *Beta vulgaris* root and leaves from Jos, Plateau State (latitude 80°24′ N and longitude 80°32′), *Persea americana* seeds from Ado-Ekiti, Ekiti State (latitude 7.6124° N and longitude 5.2371° E), and *Syzygium aromaticum* from Omu-Aran, Kwara State (latitude 8.1402° N and longitude 5.0963° E).

The herbaria were prepared and identified with the help of a taxonomist from the Forestry Research Institute of Nigeria (FRIN), Ibadan. The identification sample of each collected medicinal plant was deposited in the herbarium department of FRIN (FHI 114105, FHI 113162, and FHI 114106). The plant samples were immediately rinsed with water and cut into small pieces. After that, the samples were completely air-dried. To determine whether the samples still contained moisture, a weight variation test was performed at various intervals. Following drying to a constant weight, the samples were ground into a powder using a grinder and sieved.

### 2.2. Flavonoid-Rich Extraction and Crude Drug Formulations

Fifty (50) grams of each powdered sample of *B. vulgaris* leaf, *Persea americana* seeds, *Beta vulgaris* root, and *Syzygium aromaticum* were steeped in methanol at a concentration of 80% for 72 h to obtain a crude methanolic extract. Flavonoid-rich extraction was performed following a method described elsewhere [16]. In addition, two formulations were created by combining ground crude extracts high in flavonoids at the same ratios, as indicated in Table 1. The obtained crude formulations A and B were used for further experiments.

#### 2.2.1. Analysis of Crude Formulations A and B Using High-Performance Liquid Chromatography (HPLC–DAD)

HPLC-DAD asnalysis of crude formulations A and B was performed following methods described elsewhere [16,17].

#### 2.2.2. Determination of the Cholinesterase Activity of Crude Formulations A and B

The inhibitory effects of crude formulations A and B, which are rich in flavonoids, on acetylcholinesterase (AChE) and butyrylcholinesterase (BChE) activities were assessed using Ellman’s method [18].

### 2.3. Ex Vivo Studies

#### 2.3.1. Animals and Preparation of the Brain

Male healthy Wistar rats weighing between 150 and 200 g each were procured from the Department of Biochemistry, Bowen University, Nigeria. Brain preparation was performed following the method described by [16]. The study adhered to the approved protocols of the Bowen University Research Ethics Committee (approval number: BUI/BCH/2024/0005), which were reported using the ARRIVE guidelines.

#### 2.3.2. Ex Vivo Induction of Brain Injury

Using Fe^2+^, brain injury was induced ex vivo using the techniques outlined by Erukainure et al. [19] and Ojo et al. [16].

#### 2.3.3. Evaluation of the Activity of MAO, Also Called Monoamine Oxidase

The monoamine oxidase (MAO) activity was assessed using the method described by Green and Haughton [20].

## 3. Network Pharmacology and Molecular Dynamics Studies

### 3.1. Exploring Flavonoid-Rich Extracts of Crude Formulations A and B for Possible Target Genes for Biologically Active Compounds

The components in the flavonoid-rich extract of crude formulations A and B, which were identified as potential targets by HPLC, were predicted using SwissTarget Prediction (http://www.swisstargetprediction.ch/, retrieved on 12 July 2024) [21] while using a probability filter above zero in the “Homo sapiens” mode [22] and PharmMapper (http://www.lilab-ecust.cn/pharmmapper/, retrieved on 12 July 2024). The structures of these molecular bioactive compounds were obtained in SMILES format from the PubChem database (https://pubchem.ncbi.nlm.nih.gov/), which was accessed on 12 July 2024 [23], and uploaded to the servers. We used the species Homo sapiens to filter the findings in the SwissTarget Prediction platform, and only targets with a probability of ≥0.1 were considered. Hit target pharmacophore models, Human Protein Targets Only, and Maximum Generated Conformations 300 were the parameters utilized in PharmMapper. The models were sorted by normalized fit score, with scores below 0.5 being eliminated. Finally, the UniProt database (https://www.uniprot.org/, accessed on 12 July 2024) was used to acquire the unique matching five gene names and UniProt IDs [24].

### 3.2. Building the AD Target Database

The genes linked to AD were acquired by means of the DisGeNet database (https://www.disgenet.org/, retrieved on 12 July 2024) [25], the MalaCards database (https://www.malacards.org/, retrieved on 12 July 2024) [26], and the Online Mendelian Inheritance in Man (OMIM, https://www.omim.org/) database retrieved on 12 July 2024) [27]. “Alzheimer’s Disease” was used as the search term. One of the largest public databases of genes and variations linked to human diseases may be found on the discovery site DisGeNET. The inclusion of targets with a score of ≥0.1 was considered. Using the UniProt database, we succeeded in discovering the final gene list and establishing standard names after merging the three acquired targets and eliminating duplicate targets. (https://www.uniprot.org/, accessed on 12 July 2024) [24].

### 3.3. Identification of Bioactive Compound Targets for AD

Once all of the targets associated with AD and the active ingredients in the bioactive compounds were integrated, the overlapping targets for additional analysis were extracted using the VENNY 2.1 platform of the R language package (https://bioinfogp.cnb.csic.es/tools/venny/ accessed on 26 March 2024).

### 3.4. Construction of Bioactive Compounds from the Flavonoid-Rich Extracts of Crude Formulations A and B and the AD Target Network

Using the STRING database (https://string-db.org/, retrieved on 12 July 2024) [28], we constructed a protein–protein interaction network, limiting its scope within the genus Homo sapiens with a confidence level above 0.9 [28], a graphical user interface that is freely accessible and can be used to import, explore, and analyze biomolecular interaction connections visually. The nodes in the network represented the active substances and their target genes, while the edges displayed the interactions between the active substances and their target genes. We analyzed the network using the CytoHubba plug-in [29], which provides an easy-to-use interface for identifying important nodes in biological networks.

### 3.5. Pathway and Functional Enrichment Analysis

Gene Ontology (GO) and Kyoto Encyclopedia of Genes and Genomes (KEGG) enrichment analyses were used to evaluate the intersecting genes using the Shiny GO 0.77 tool (http://bioinformatics.sdstate.edu/go/, retrieved on 12 July 2024) [30]. The top ten results are displayed with a cutoff value of *p* < 0.05 and an FDR of <0.05.

### 3.6. Molecular Docking Analysis

Based on the results of the PIP-network interaction, important targets were selected for molecular docking analysis. Although MOA, which had a far lower score, was considered because it formed a strong connection with both AChE and BChE and has a great deal of potential as a viable AD treatment target, AChE and BChE were chosen because they possessed significantly higher values in the PPI network.

### 3.7. Molecular Docking Studies of Bioactive Compounds Against Target Substances

#### 3.7.1. Preparation of Protein Structure

The three target protein 3D structures that were obtained from the Protein Data Bank (http://www.rcsb.org, accessed on 26 March 2024) are human monoamine oxide B complexed with safinamide (PDBID: 2V5Z), human acetylcholinesterase (hAChE) complexed with donepezil (PDBID: 4EY7), and human butyrylcholinesterase (hAChE) complexed with decamethonium (PDBID: 6EP4). Using MGL-AutoDockTools (ADT, v1.5.6), missing hydrogen atoms were added to all crystal structures, while the existing ligands and water molecules were eliminated [31].

#### 3.7.2. Ligand Preparation

The HPLC-detected phytocompounds from the flavonoid-rich extract of crude formulations A and B as well as the structure data format (SDF) of the reference inhibitors (donepezil, decamethonium, and safinamide) were retrieved from the PubChem database (www.pubchem.ncbi.nlm.nih.gov, accessed on 26 March 2024). Open Babel was utilized to further transform the chemicals into the pdb chemical format [32]. The carbon atoms were combined with nonpolar hydrogen molecules, and Gasteiger-type polar hydrogen charges were assigned to the atoms. Additionally, AutoDock Tools was utilized to convert the ligand molecules into the dockable PDBQT format.

#### 3.7.3. Molecular Docking Protocol Validation

The extracted crystallized ligand from the two proteins was superimposed with the docked poses of the cocrystallized ligands (donepezil) that had the lowest binding affinity according to the docking evaluation to verify the procedure employed for the docking evaluation. This procedure was used to validate the docking methodology used for the docking of the bioactive substances identified by HPLC. Using Discovery Studio Visualizer (BIOVIA discovery studio, 2020), the RMSD was calculated following the superimposition.

#### 3.7.4. Molecular Docking of Phytochemicals with Targeted Active Sites

Using AutoDock Vina integrated into PyRx 0.8, active site target molecular docking of the reference inhibitors and the HPLC-DAD-discovered compounds to the binding site of the three target proteins was carried out [33]. The bioactive chemicals were imported using Open Babel and integrated into PyRx 0.8 prior to docking analysis [32]. Moreover, Open Babel further reduced the number of bioactive components. The universal force field (UFF) and the optimization procedure were utilized as the energy minimization parameter and conjugate gradient descent, respectively. Mapping the amino acid residues surrounding the natural ligand binding site allowed the identification of the binding site coordinates of the target proteins. Table 2 displays the dimensions of the grid boxes that were created around the targets’ actives. Discovery Studio Visualizer version 16 was used to perform an interactive analysis on the chosen conformer from the docking analysis.

#### 3.7.5. Molecular Dynamics

For a 100 ns molecular dynamics simulation, the complexes of the top two phytochemicals with 4ey7 and 1b2y were also determined. The GROMACS 2019.2 and GROMOS96 43a1 force fields were used in the investigation. Charmm GUI was used to create topology files for the proteins and ligands [16,34,35]. The solvation system, periodic boundary conditions, physiological conditions, system minimization, and equilibration under constant number of atoms, pressure, and temperature (NPT) in the simulation were consistent with those employed in our previous study [16,36,37,38]. The temperature was maintained at 310 K, and the pressure was maintained at 1 atm using a Parrinello–Rahman barostat and velocity rescales. A leap-frog integrator was utilized with a 2-femtosecond time step. Every system had a 100 ns simulation, during which 1000 frames—a snapshot every 0.1 ns—were captured for every system. H-bonds, ROG, SASA, RMSD, and RMSF were obtained from the MD trajectories.

#### 3.7.6. Calculation of the Binding Free Energy with MM-GBSA

The Molecular Mechanics Generalized Born Surface Area (MM-GBSA) method, along with decomposition analysis using the gmx MMPBSA package, was employed to obtain the binding energies of amino acids within 0.5 nm of the ligand. This analysis aimed to determine the binding free energy of the two top-docked phytochemicals from the initial docking study [39,40]. The techniques employed were the same as those described in our earlier reports [16,37,38].

#### 3.7.7. Data Analysis

The data were analyzed using one-way ANOVA, and the mean ± standard deviation (*n* = 3) represents the output. We subsequently used Tukey’s multiple range post hoc test to determine statistically significant differences (*p* < 0.05). For graph charting, GraphPad Prism version 9.0 was used.

## 4. Results

### 4.1. HPLC-DAD Analyses of Flavonoid-Rich Extracts of Crude Formulations A and B

HPLC-DAD analysis of crude formulation A revealed the presence of six (6) bioactive flavonoids, namely, caffeic acid, syringic acid, gallic acid, kaempferol, quercetin, and rutin, while Form B contained seven bioactive flavonoids, namely, myricetin, rutin, gallic acid, caffeic acid, quercetin, p-coumaric acid, and methyl gallate (Table 3, Figures S1 and S2).

### 4.2. Inhibitory Action of Butyrylcholinesterase and Acetylcholinesterase

**Figure 1:**
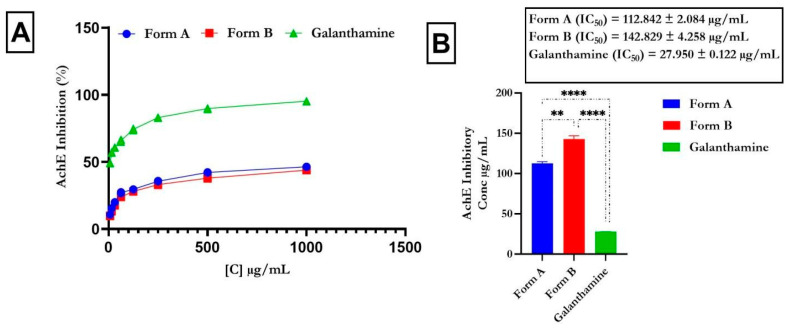
Effect of flavonoid-rich crude drug formulations (**A**,**B**) on acetylcholinesterase activity Legend: The mean ± SD (*n* = 3) is used to describe the data; *t* tests show that *p* < 0.0001. Standard medication: galantamine. ** is significant at *p* < 0.01 when Form A is compared to form B; **** is significant at *p* < 0.0001 when Form A and B is compared to galantamine.

**Figure 2:**
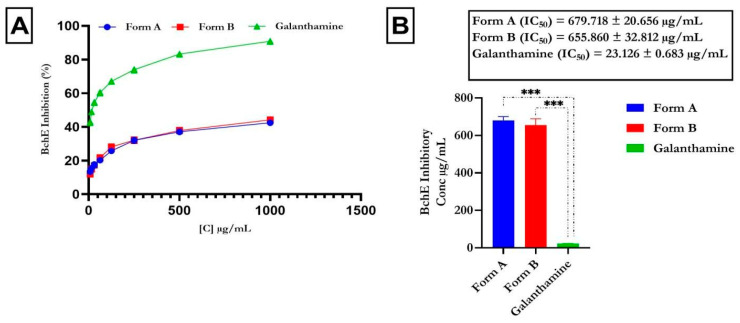
Effect of flavonoid-rich crude drug formulations (**A**,**B**) on butyrylcholinesterase activity Legend: The mean ± SD (*n* = 3) is used to describe the data; *t* tests show that *p* < 0.001. Standard medication: galantamine. *** is significant at *p* < 0.001 when Form A and B is compared to galantamine.

We assessed the potential of the formulations to inhibit AChE and BChE, pivotal enzymes in modulating acetylcholine levels crucial for nerve transmission. The percentage inhibition at varying concentrations is depicted in Figure 1 and Figure 2, illustrating the dose-dependent inhibitory effects on both enzymes. The flavonoid-rich extracts of crude formulations A (IC_50_ = 112.842 ± 2.084 µg/mL) and B (IC_50_ = 142.829 ± 4.258 µg/mL) exhibited notable inhibitory activity (*p* < 0.0001) against AChE, despite being less potent than the typical control, galantamine (IC_50_ = 27.950 ± 0.122 µg/mL), as shown in Figure 1. Similarly, the flavonoid-rich extracts of crude drug formulations A (IC_50_ = 679.718 ± 20.656 µg/mL) and B (IC_50_ = 655.860 ± 32.812 µg/mL) had notable (*p* < 0.0001) inhibitory effects on BChE, although the effects were weaker than those of galantamine (IC_50_ = 23.126 ± 0.683 µg/mL), as indicated in Figure 2.

### 4.3. Activity of Monoamine Oxidase

**Figure 3:**
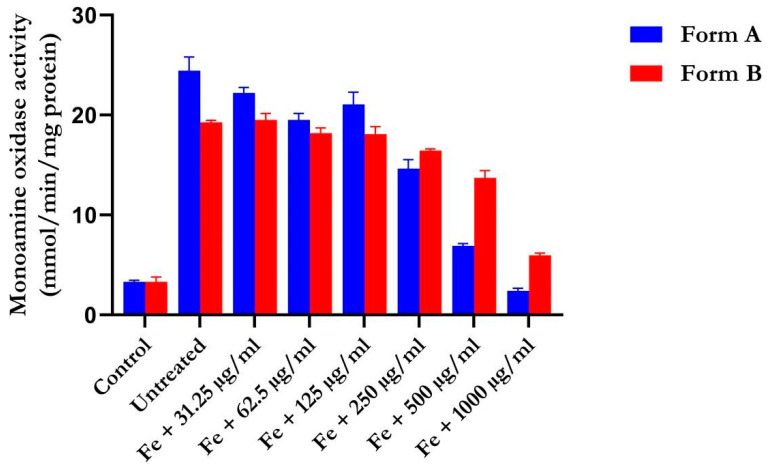
Effect of different concentrations of flavonoid-rich crude drug formulations A and B on the activity of rat brain monoamine oxidase (MAO) ex vivo. There was a significant decrease in monoamine oxidase activity in the range of extracts rich in flavonoids from crude drug formulations A and B when compared to the group induced exclusively with FeSO_4_. Legend: the mean ± SD (*n* = 3) is used to describe the data.

The inhibitory effects of flavonoid-rich crude drug formulations A and B on monoamine oxidase activity (MAO) in the oxidized brain were evaluated, and the results are shown in Figure 3. The experiment showed that the untreated rats had increased MAO activity (*p* < 0.0001). However, we observed a notable decrease in rat brains treated with various doses of the formulations (*p* < 0.0001). The inhibitory effect of the extracts rich in flavonoids from crude drug formulations A and B on enzyme activity was concentration-dependent; the effect increased as the concentration of the crude formulations decreased, with the greatest inhibitory effect recorded at 1000 µg/mL.

### 4.4. Screening of Bioactive Compounds and Databases for AD Targets

**Figure 4:**
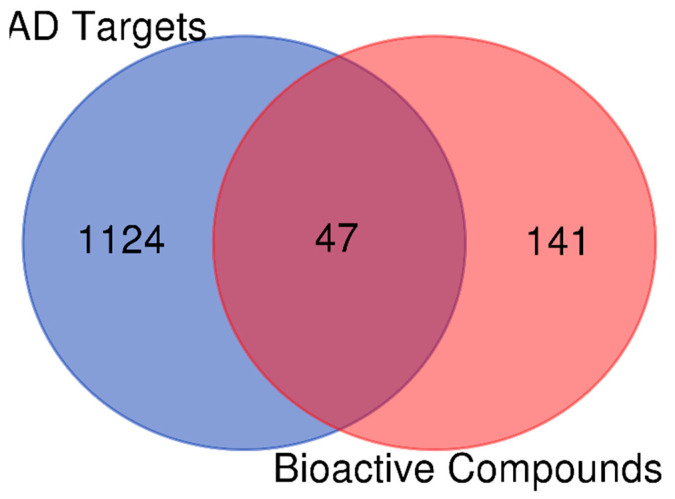
Venn diagram showing the genes linked to AD and the bioactive chemicals from flavonoid-rich crude drug formulation A- and B-associated targets. Forty-seven genes related to both Alzheimer’s disease (AD) and C. sativa were identified.

The screening of gallic acid, caffeic acid, syringic acid, rutin, quercetin, kaempferol, methyl gallate, p-coumaric acid, and myricetin in the SwissTarget and PharmMapper databases revealed 188 targets, while after the duplicates were removed, we obtained 1171 genes associated with AD from the DisGeNet, MalaCards, and Online Mendelian Inheritance in Man Databases. Using a Venn diagram, common AD genes and targets associated with bioactive compounds were identified. Forty-seven putative anti-AD genes were chosen and regarded as important targets. This schematic is displayed in Figure 4.

### 4.5. Analysis of the Target Protein‒Protein Interaction Network

**Figure 5:**
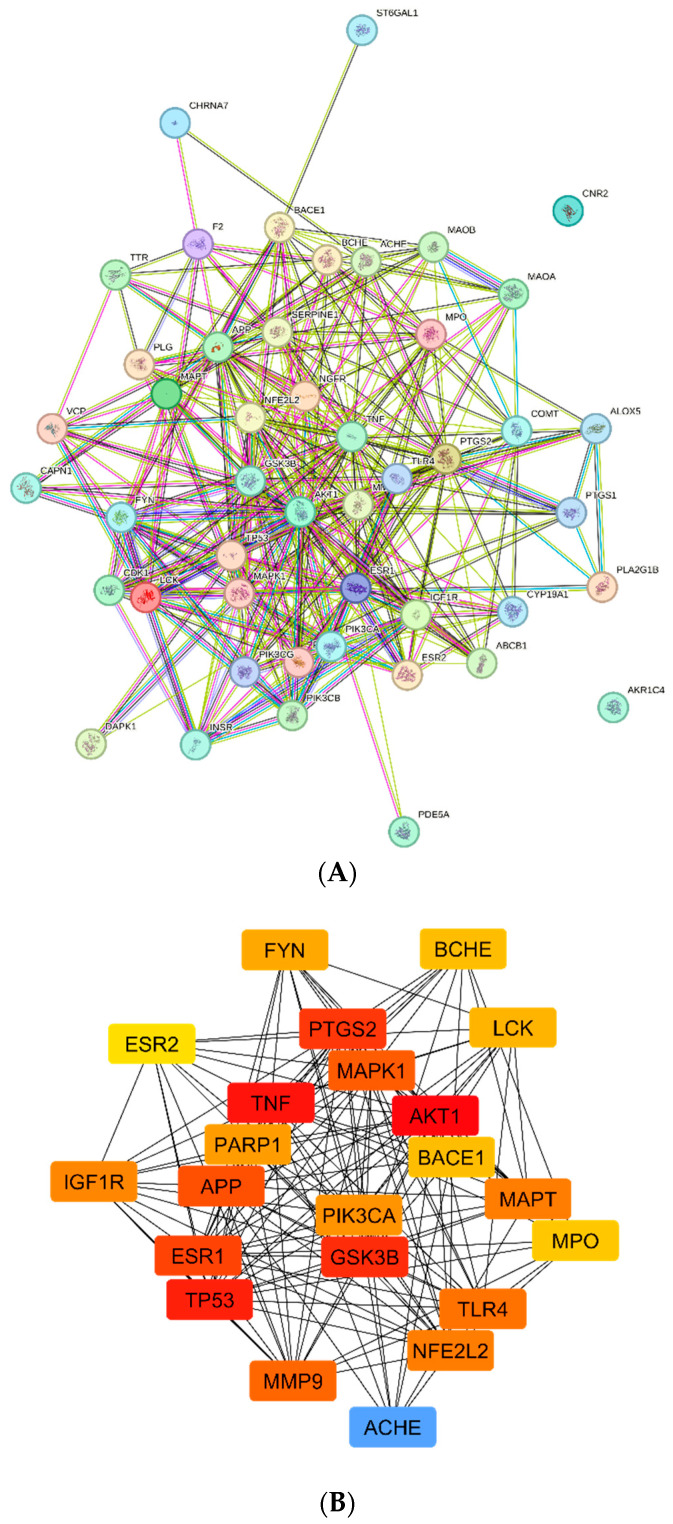
PPI network of target and AD-related genes created in Cytoscape utilizing (**A**,**B**) the STRING database and (**B**) the core key subnetwork of the top 22 nodes examined by CytoHubba.

Figure 5 displays the diagrammatic form of the PPI network. There were 326 edges and 47 nodes, with an average node degree of 13.9. The network was then loaded into Cytoscape 3.8.2 to identify important subnetworks. The network clustering process was carried out using proximity and degree metrics based on the scores determined by CutyoHubba and CytoNca. According to the values of genes that were greater than the median across all the results, the top 22 targets were filtered out. The top five targets are the PIK3CA, APP—amyloid-beta precursor, AKT1 GSK3B, and TLR4 proteins. Also, among the several targets that were identified, AChE, BChE, and MAO, which were ranked as 10, 14, and 34 based on the closeness parameter, respectively. These proteins have been identified to be inhibited by the formulation in the in vitro *assays* that were used to validate the activities of the formulations; hence, they were selected for further analysis.

### 4.6. Enrichment Analysis of Overlapping Targets

**Figure 6:**
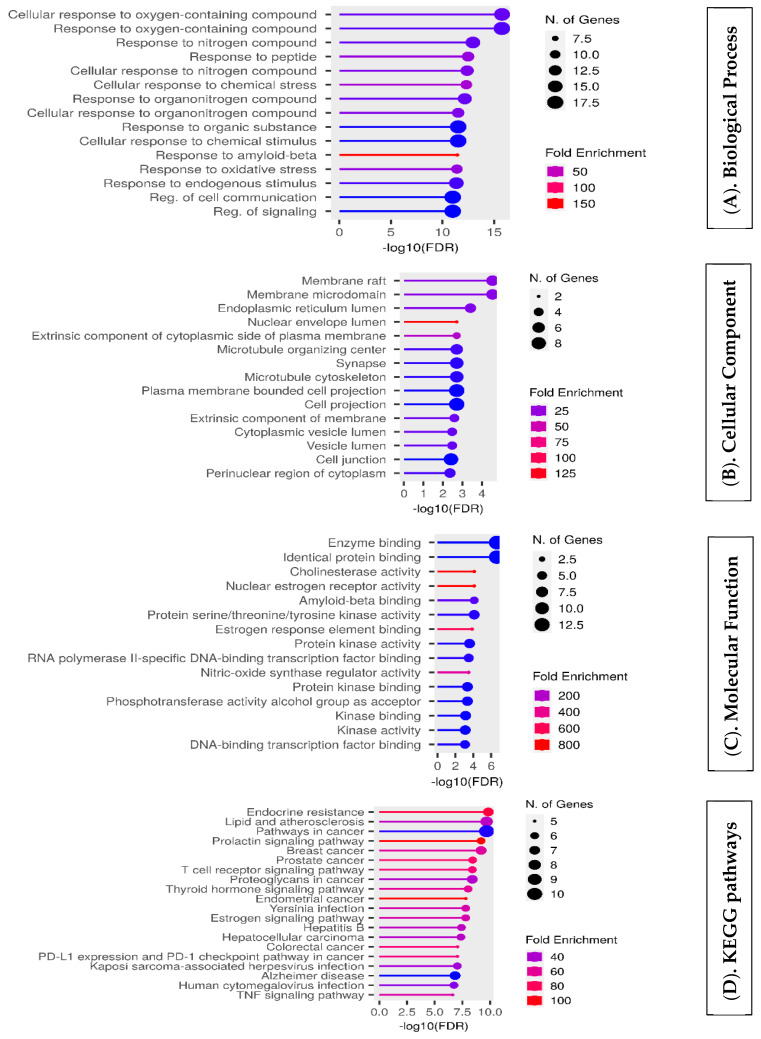
Representation of functional annotation and enriched pathways. (**A**) GO biological processes. (**B**) Cellular components of GO. (**C**) Molecular function of GO. (**D**) KEGG pathway analysis.

Gene Ontology (GO) enrichment revealed the top 22 out of the 47 overlapping targets for each enrichment. According to our biological process (BP) analysis (Figure 6A), the functions of the bioactive compounds were primarily focused on the response to compounds containing oxygen and the response to peptides containing oxygen. The majority of the genes encoded proteins in the endoplasmic reticulum lumen, cell membrane ratio and microdomains, mitochondria, and, notably, synapses (Figure 6B). The molecular function (MF) elements (Figure 6C) primarily included identical proteins, binding to enzymes, cholinesterase and nuclear estrogen receptor activity, and, notably, amyloid-beta binding. The bioactive compounds were primarily linked to endocrine resistance, lipids, cancers, and most notably, AD, according to Kyoto Encyclopedia of Genes and Genomes (KEGG) pathway enrichment analysis (Figure 6D).

### 4.7. Molecular Docking of Bioactive Compounds with Important Targets

**Figure 7:**
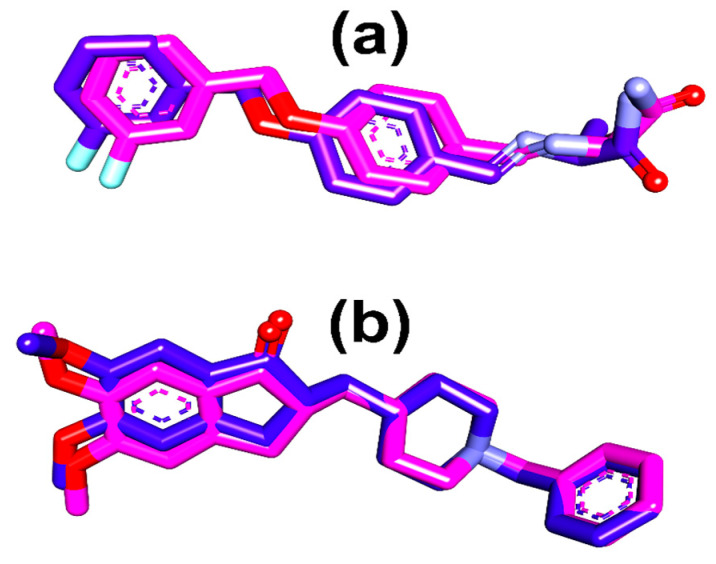
Superimposition of selected conformers from the docking analysis of the native ligand on the extracted conformation of (**a**) donepezil and (**b**) safinamide. The blue compounds represent the docked conformer, while the red conformation represents the conformation extracted from 4EY7 and 2V5Z.

**Figure 8:**
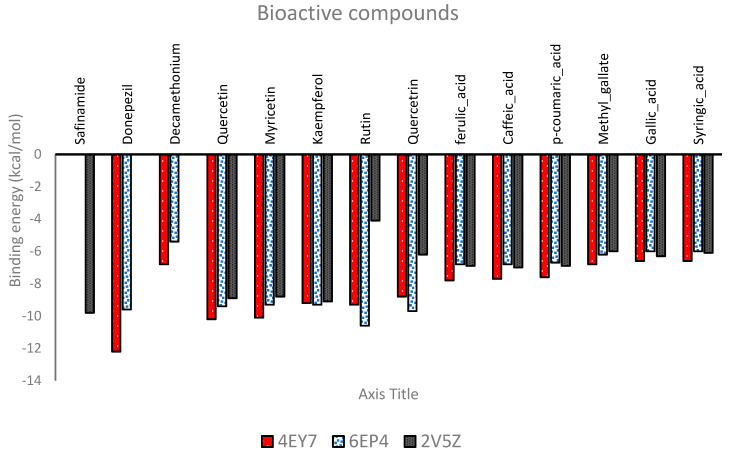
Binding energies of bioactive compounds from flavonoid-rich extracts of crude drug formulations A and B that were identified by HPLC against target proteins.

We used molecular docking based on the PPI network to assess whether AD-related core targets and bioactive compounds could bind. The basis for the selection of target proteins stems from their enrichment in the PPI and related to the prior in vitro assay that was used to validate the activity of the formulation. Prior to the bioactive compounds being docked to the target proteins’ active sites, the process was verified, and the superimposed docked conformations of donepezil and safinamide were superimposed against the crystalized conformation. The computed RMSD for both compounds were 0.4642 and 1.0496 Å, respectively (Figure 7). The binding energies of the bioactive compounds with respect to the three target proteins are shown in Figure 7. Donepezil, decamethonium, and safinamide, the native ligands of the 4EY7, 6EP4, and 2V5Z targets, presented binding energies of −12.2, −5.4, and −9.9 kcal/mol, respectively. The two compounds with the lowest binding energies to the targets were chosen. Quercetin (−10.2 kcal/mol) and myricetin (−10.1 1 kcal/mol) are the top two compounds for 4EY7; rutin (−10.6 kcal/mol) and quercetin (−9.7 kcal/mol) are for 6EP4; and kaempferol (−9.1 kcal/mol) and quercetin (−8.9 kcal/mol) are for 2V5Z. The top two compounds of 6EP4 had lower binding energies than did the reference compound (decamethonium, −5.4 kcal/mol), but the binding energies for the 4EY7 and 2V5Z targets were rather close to those of the reference compounds. Moreover, at least two of the protein targets showed repeated high binding inclinations to quercetin, and rutin 16 (Figure 8).

### 4.8. Amino Acid Interactions of the Top Two Compounds from the Docking Analysis and Reference Molecules with the Five Protein Targets

**Figure 9:**
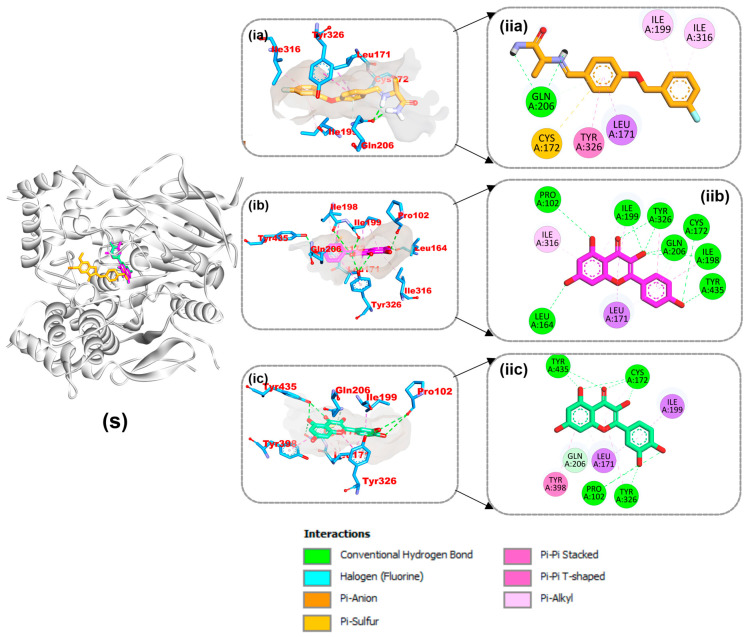
Interactions of top-ranked compounds and the reference inhibitor (donepezil) with amino acids in the 4EY7 active site. Sticks are used to represent the ligands. (**ia**,**ib**,**ic**) Three dimensional interactions, (**ii**) two dimensional interactions, and (S) cartoon representation showing ligands in the active site (**a**) Donepezil, (**b**) myricetin, and (**c**) quercetin.

**Figure 10:**
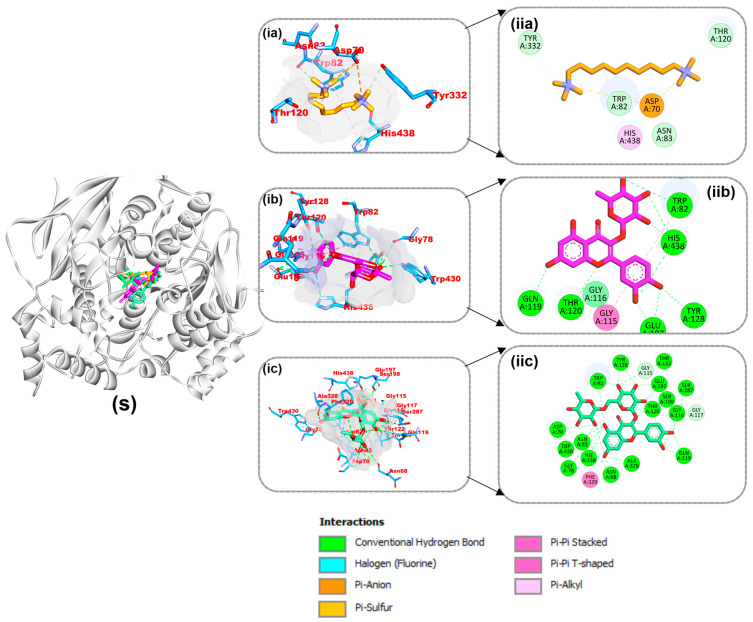
Interactions of top-ranked compounds and reference inhibitor (decamethonium) with amino acids in the 6EP4 active site. Sticks are used to represent the ligands. (**ia**,**ib**,**ic**) Three dimensional interactions, (**ii**) two dimensional interactions, and (S) cartoon representation showing ligands in the active site (**a**) Decamethonium, (**b**) rutin, and (**c**) quercetin.

**Figure 11:**
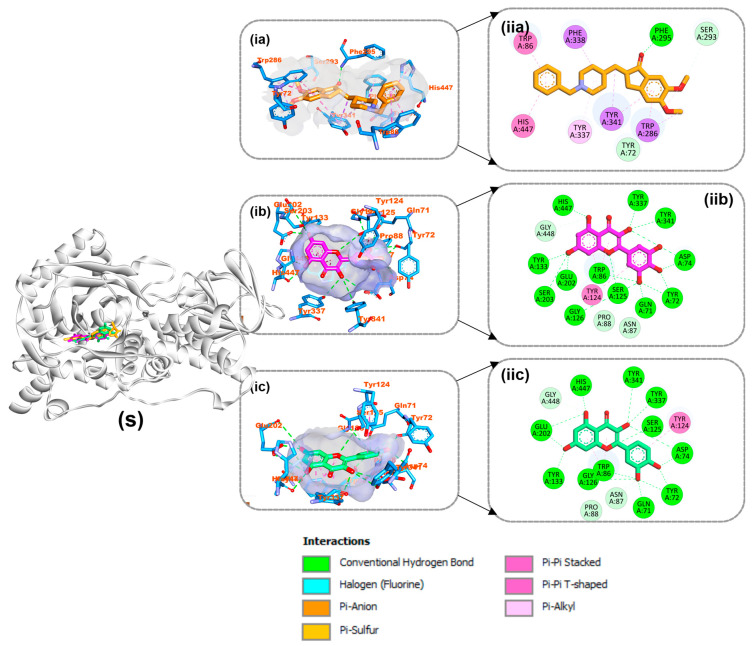
Interactions of top-ranked compounds and reference inhibitor (Safinamide) with amino acids in the 2V5Z active site. Sticks are used to represent the ligands. (**ia**,**ib**,**ic**) Three dimensional interactions, (**ii**) two dimensional interactions, and (S) cartoon representation showing ligands in the active site. (**a**) Safinamide, (**b**) kaempferol, and (**c**) quercetin.

The connections between the reference molecule and the top two compounds from the docking study with residues from the target protein binding site are shown in Figure 9, Figure 10 and Figure 11. A validation study revealed that donepezil was stretched in the long, narrow, and hydrophobic gorge of 4ey7 in a binding configuration similar to that of the natural ligand. There was a single hydrogen bond between Phe295 and the carbonyl oxygen of the indenone ring. Two Pi-alkyl interactions were generated by Tyr337 and Tyr341 of 4ey7 with the donepezil piperidine ring. With Donepezil’s 1-benzyl unit, Trp86 and His447 participate in an aromatic pi-pi stacking interaction. It was discovered that the 5-methoxy unit of inden-1-one and the piperidine rings Trp286 and Phe338 have two pi-sigma bonds (Figure 9).

As shown in Table 4, myricetin and quercetin, the best bioactive compounds docked to the 4EY7 target, were docked similarly to each other in the gorge’s active site, generating many hydrogen bonds and hydrophobic contacts. The interaction of myricetin in the active site was stabilized by 12 hydrogen bonds, 3 carbon hydrogen bonds, 2 pi-pi T shapes, and pi-pi stacking, while quercetin was stabilized by 12 hydrogen bonds, 3 carbon hydrogen bonds, 2 pi-pi T shapes, and pi-pi stacking. Decamethonium was stabilized in the active site of 6EP4 with major hydrophobic interactions such as pi-alkyl interactions with His438 and Trp82 and an attractive charge with Asp70 without hydrogen bonds. Quercetin and rutin, on the other hand, were stabilized by several hydrogen and hydrophobic contacts, including pi-sigma (Ala328), pi-pi stacking (Phe329), and pi-pi T-shaped contacts with Trp430 of 6EP4 (Figure 10). The reference compound for 2V5Z, safinamide, was stabilized with catalytic residues through several hydrophobic contacts, including pi-sigma (Leu171), pi-sulfur (Cys172), and pi-pi T-shaped interactions and one hydrogen bond with Gln206 of 2V5Z. In addition, the two top docked bioactive compounds produced multiple hydrophobic interactions and additional hydrogen bonds with the catalytic residues. Kaempferol forms eight hydrogen bonds with 2V5Z and hydrophobic contacts, including pi-sigma (Leu171 and Ile199), pi-pi T-shaped (Tyr326), amide-pi stacking (Ile198), and pi-alkyl (Ile316, Leu171 and Ile199) interactions. Quercetin was stabilized with four hydrogen bonds in the active site of 2V5Z and several hydrophobic contacts, including pi-sigma (Ile199 and Leu171), pi-pi T-shaped (Tyr398), and pi-pi stacking (Tyr326) (Figure 11).

### 4.9. Molecular Dynamics Simulations

**Figure 12:**
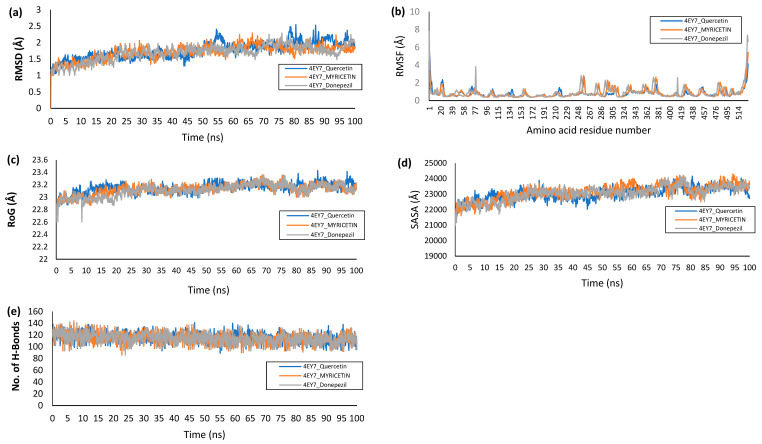
Plots of thermodynamic parameters computed from the analysis of the MD trajectories of 4EY7 complex systems: (**a**) Backbone-Root Mean Square Deviation (RMSD), (**b**) per residue Root Mean Square Fluctuations (RMSF), (**c**) radius of gyration, (**d**) Surface Accessible Surface Area (SASA), and (**e**) number of hydrogen atoms.

To compare the thermodynamic stability of the top two bioactive compounds complexed with the 4EY7 protein with that of the reference chemical-bound system, the number of intramolecular hydrogen bonds as well as the RMSD, ROG, and SASA were assessed during the simulation. Table 5 displays the averages and standard deviations of all thermodynamic parameter values, while Figure 12 displays the parameter’s time-dependent spectra. The RMSD trajectory illustrates how a complex’s structure deviates over time from its unbound structure. Generally, an RMSD value of less than 3 Å is acceptable [41]. The 4EY7 complex RMSD graphs indicated equilibrium before 10 ns, and for the duration of the run, the system fluctuated very little (Figure 12a). By measuring the RMSF, the thermodynamic flexibility of amino acid residues following the binding of myricetin and quercetin to the 4EY7 active site was examined (Figure 12b).

During an MD simulation, the ROG, another thermodynamic stability metric, measures a complex’s time-dependent compactness; the lower the value is, the more stable and compact the complex is. The complex systems of the top-docked compounds presented similar mean ROG values to those of donepezil (23.20 Å, 23.19 Å, and 23.22 Å) (Figure 12c). It is commonly assumed that the binding of the top-docked compounds significantly impacts the structural integrity of the protein, which could lead to the unfolding of the protein structure. A strong positive correlation between the RMSD and ROG usually indicated a distortion of the protein structure. In this study, even though a higher RMSD value was recorded, a close mean ROG value was reported for the top docked compounds compared to the reference compound (donepezil).

The SASA is a measure of protein folding and surface area changes during a simulation; greater SASA values imply an increase in protein volume ([42]). Along with the mean SASA values of donepezil, those of the top-docked complexes were similar. The findings from the SASA analysis corroborate those of the mean ROG, indicating that the binding of the top-docked compound did not cause unfolding of the protein (Figure 12d). The average number of H-bonds created in the molecules as a whole did not vary significantly during the experiment. A close number of hydrogen bonds were observed in the ligand-bound complexes (Figure 12e).

### 4.10. Molecular Mechanics Generalized Born Surface Area (MM-GBSA) Analysis

**Figure 13:**
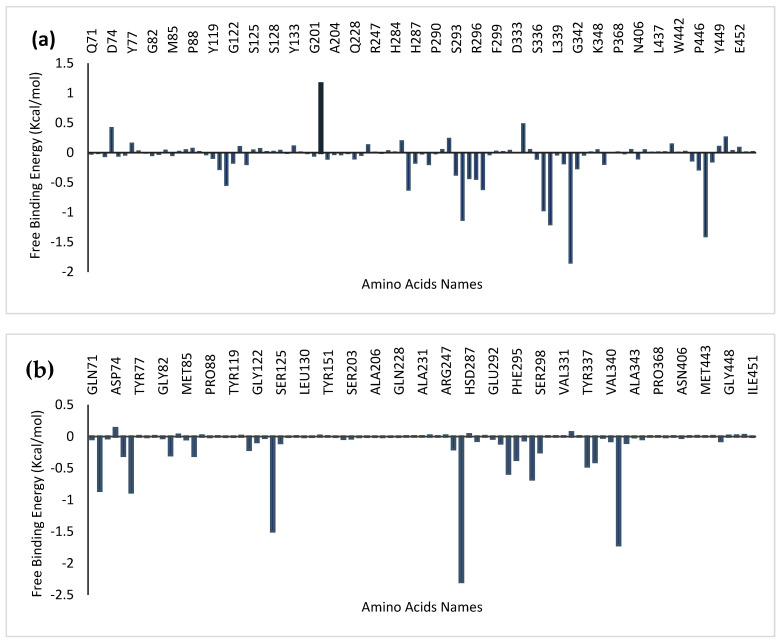

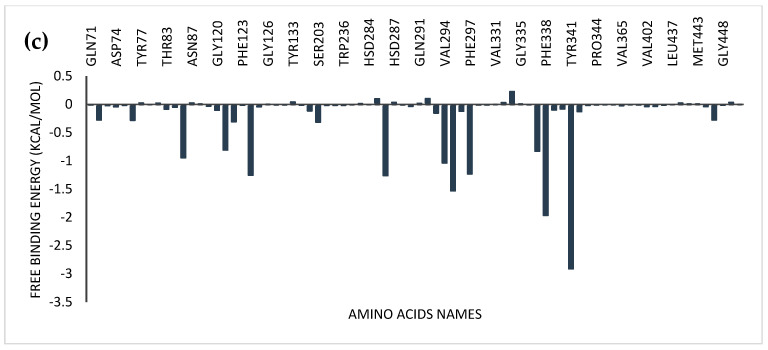
Plot of the Molecular Mechanics Generalized Born Surface Area (MM-GBSA) showing the amino acid residues of hAChE that contribute to the total binding free energy of (**a**) donepezil, (**b**) quercetin, and (**c**) myricetin.

The binding free energy of the two topmost phytochemicals docked to the protein 4ey7 was determined using the MMGBSA technique. Among the top-docked compounds, myricetin had the highest negative binding free energy of −25.78 ± 4.04 kcal/mol to 4EY7; this binding energy was greater than that of donepezil (−19.92 ± 3.62). Notably, the binding free energies of both phytocompounds are greater than those of donepezil. Table 6 lists the different parts that add up to the overall binding free energy. Using decomposition analysis, the contributing amino acids that make up the overall binding energy were examined and are shown in Figure 13. The majority of the binding free energy was attributed to the interacting residues during static docking.

## 5. Discussion

Many Alzheimer’s patients are currently interested in complementary or alternative therapies that use readily available herbal products. In the interim, a variety of elements have a direct impact on the selection criteria for herbal formulations used in illness treatment. The stage at which the disease progresses, the kinds of comorbidities that are present, the accessibility and cost of the herbs, and their safety profile are all taken into consideration when choosing herbal treatments for Alzheimer’s disease [43]. Three medicinal plants were chosen for the study based on these facts as well as the use of herbal medicines for their neuroprotective properties by different indigenous and ethnic groups in different parts of Nigeria.

The medicinal value of the majority of plants is attributable to their respective secondary metabolites. Secondary metabolites are organic substances found in plants that are produced from primary metabolites and have chemically distinct structures. These secondary metabolites primarily belong to the following classes: phenolics, terpenoids, alkaloids containing nitrogen, and substances containing sulfur [44]. Secondary metabolites found in plants are important radical scavengers. According to a study by Yu-Jie et al. [45], the two primary MD simulation categories of antioxidant phytochemicals are carotenoids and polyphenols. The five types of dietary and herbal polyphenols are as follows: tannins, coumarins, stilbenes, phenolic acid, and flavonoids [45]. Additional classifications for flavonoids include isoflavonoids, anthocyanidins, flavonols, flavones, flavanols, flavanones, and flavonols, all of which have been linked to antioxidant properties.

Acetylcholinesterase (AChE) and butyrylcholinesterase (BChE) are enzymes that degrade acetylcholine, leading to reduced neurotransmission and progressive cognitive decline. Inhibiting these enzymes can enhance cholinergic transmission, thereby alleviating Alzheimer’s disease (AD) symptoms, including memory loss, and reducing mortality risk [46]. Consequently, cholinesterase inhibitors are currently the only approved therapy for AD and neurodegenerative dementia [47]. Cholinergic dysfunction is marked by increased AChE activity, making it a viable target for therapeutic interventions [48]. Additionally, AChE inhibition not only improves cholinergic signaling but also reduces amyloid-beta peptide formation and aggregation in AD [49]. In our study, the flavonoid-rich crude formulations A and B exhibited notable inhibition of AChE and BChE, suggesting their potential neuroprotective effects on the management of AD. This inhibition implies the ability of the crude drug formulations to mitigate AD-related enzyme activity, thereby showing promise as therapeutic agents. Our findings are consistent with previous reports [16].

The inhibition of monoamine oxidase (MAO) is a crucial biomarker in the management of AD. The ability of the flavonoid-rich crude formulations A and B to inhibit this enzyme suggests potential therapeutic benefits for AD. This reduction in MAO activity, attributed to the crude drug formulations, could increase the levels of amine neurotransmitters such as dopamine and serotonin, while also preventing the degradation of amines by reactive oxygen species (ROS) [50].

Our study provides insights from the combination of molecular docking, network pharmacology, and MD to assess the possible active ingredients of flavonoid-rich crude drug formulations A and B against AD in intricate processes. The screening results revealed 1171 genes linked to AD and 188 AD targets from the DisGeNet, MalaCards, and Online Mendelian Inheritance in Man databases. Through 47 potential anti-AD gene targets, the “active ingredient-target” interaction network and Venn diagram allowed for an intuitive understanding of the precise relationship between crude drug formulations and AD. Eleven related active bioactive compounds were identified by our network analysis, all of which may play a relatively significant role in the anti-Alzheimic effect of crude drug formulations on AD.

A protein–protein interaction (PPI) network was constructed and then put into Cytoscape using the Search Tool for the Retrieval of Interacting Genes/Proteins (STRING) database. In this network, the 38 target proteins are nodes, and the connections between proteins are edges. The complex bioactive-target network reflected that the bioactive compounds in the flavonoid-rich crude drug formulations A and B corresponded to multiple bioactive constituents, thus revealing multicomponent properties. The target proteins with high confidence, defined as those with a score higher than 0.9, were chosen. The highest correlation suggested a strong correlation between the targeted genes, suggesting that each of these genes may be a significant target [51].

The intersection targets were subjected to GO and KEGG pathway enrichment analysis using the Shiny GO 0.77 tool. The 47 overlapping targets of the top 22 enriched targets were identified. Among the GO-enriched categories, peptides and their reactions to oxygen-containing chemicals were the primary subjects of BP enrichment. The majority of genes encode proteins in the rat cell membrane, microdomains, endoplasmic reticulum lumen, mitochondria, and, notably, synapses. Additionally, among other factors, the enhanced items in the molecular function (MF) category were primarily linked to the binding of cholinesterase, amyloid-beta, and protein kinase.

The intersecting proteins involved in the extracellular environment, such as membrane rafts, membrane microdomains, microtubule cytoskeletons, and synapses, were classified as enriched in cellular components (CCs).

Overall, our GO enrichment results showed that targets in the cytoplasm, cell membrane, or extracellular space that are closely related to the regulation of amyloid beta, the cellular response, and metabolism during the onset and progression of AD may bind to anti-AD targets of crude drug formulations. KEGG enrichment analysis was then carried out to identify possible signaling pathways involved in the effects of crude medication formulations on AD by merging genomes with cellular and species data. According to our research, endocrine resistance pathways in cancer and AD signaling pathways are the primary targets shared by AD and crude drug formulations.

We used molecular docking based on the PPI network to assess whether AD-related core targets and bioactive drugs could bind. Prior to the bioactive chemicals being docked to the target proteins’ active sites, the process was verified, and the docked conformations of donepezil and safinamide were superimposed on the crystallized conformation. For AChE (4EY7), quercetin and myricetin were the top two compounds; for BChE (6EP4), rutin and quercetin; and for monoamine oxidase A (2V5Z), kaempferol and quercetin were the top two compounds. The top two compounds of 6EP4 had higher binding energies than did the reference compound (decamethonium, −5.4 kcal/mol), but their binding energies for the 4EY7 and 2V5Z targets were rather close to those of the reference compounds.

A validation study revealed that donepezil was stretched in the long, narrow, hydrophobic gorge of 4ey7 in a binding configuration akin to that of the natural ligand. There was one hydrogen bond between Phe295 and the carbonyl oxygen of the indenone ring. Two Pi-alkyl interactions were generated by Tyr337 and Tyr341 of 4ey7 with the donepezil piperidine ring. With donepezil’s 1-benzyl unit, Trp86 and His447 participate in an aromatic pi-pi stacking interaction.

The mean RMSD values of the complexes of quercetin and myricetin fluctuated greatly, although they were still quite close to those of the reference compound donepezil. It can be deduced from the RMSD analysis of the trajectories that the binding of myricetin and quercetin did not generally affect the thermostructural stability of the protein [52]. The ability of atoms and residues in a protein structure to form stable intra- and intermolecular bonds is taken into account by the RMSF value, which measures the average fluctuation of these elements over the course of a simulation. The stronger the bonds are and the greater the affinity of the ligand for the protein is, the less fluctuation there is, especially in the active site where ligand binding and catalysis occur [53]. Compared to that of the reference inhibitor, the binding of the top-docked ligands in the present study did not result in a change in the protein residues. The greatest variation was observed for amino acid residues 243 and 375. Terminal fluctuations are the cause of the fluctuations at the end of the spectrum. In this study, the top-docked substances showed similar mean ROG values compared to the reference compound (donepezil), despite the greater RMSD value being recorded. During a simulation, the SASA measures changes in surface area and protein folding; higher SASA values indicate higher protein volumes [42]. Compared to the mean SASA values of donepezil, those of the top-docked complexes were similar. The results of the SASA analysis support the mean ROG results, suggesting that protein unfolding was not triggered by the binding of the top-docked molecule. The stability of a protein structure is mostly dependent on intramolecular hydrogen bonds and distance, which can be assessed to ascertain how ligand binding impacts a protein’s stability during simulation [41].

The binding free energy measures the energy differential between the ligand and receptor that are bound and unbound in a complex; the larger the negative value is, the greater the ligand’s affinity for the protein [41]. The binding free energy estimates offer comprehensive data on the binding mechanisms of the top-docked compounds that are critical to the preliminary stages of drug discovery and development [54].

## 6. Conclusions

This study provides compelling evidence that flavonoid-rich crude drug formulations have significant potential as therapeutic agents in the treatment of Alzheimer’s disease (AD). By integrating molecular docking, network pharmacology, and molecular dynamics (MD) simulations, we identified key bioactive compounds from the formulations that target crucial AD-related proteins. The inhibitory effects of these formulations on acetylcholinesterase (AChE), butyrylcholinesterase (BChE), and monoamine oxidase (MAO) highlight their potential in modulating cholinergic transmission and reducing oxidative stress, which are central to AD pathogenesis. The molecular interactions observed in docking studies and the stability of the compounds during MD simulations further reinforce the therapeutic promise of these formulations. These findings offer a scientific rationale for the use of selected medicinal herbs in managing AD and underscore the importance of exploring natural compounds as viable alternatives to conventional AD treatments. The top enriched targets that were not considered in the presented studies are suggested for further study.

## Figures and Tables

**Table 1:** Ratio of crude extracts high in flavonoids utilized during formulation.

	*Ratio of Crude Extracts High in Flavonoids*	
*Plants*	*Form A*	*Form B*
*B. vulgaris leaf*	*2.5*	*
*Persea americana seeds*	*	*2.5*
*Beta vulgaris root*	*	*2.5*
*Syzygium aromaticum*	*2.5*	*

* Not part of formulation.

**Table 2:** The target enzyme binding site coordinates.

*Dimensions*	*6ep4 (Å)*	*2v5z (Å)*	*4ey7 (Å)*
*center_x*	*−19.83*	*51.92*	*−13.35*
*center_y*	*−41.39*	*155.81*	*−43.63*
*center_z*	*47.10*	*28.67*	*27.41*
*Size x*	*15.81*	*13.96*	*19.75*
*Size y*	*17.42*	*14.53*	*13.23*
*Size z*	*14.59*	*14.59*	*16.84*

**Table 3:** Bioactive compounds identified in crude drug formulations.

*Compounds*			
*Form A*	*Conc (mg/mL)*	*Form B*	*Conc (mg/mL)*
*Gallic acid*	*6.68*	*Myricetin*	*4.63*
*Caffeic acid*	*8.92*	*p-coumaric acid*	*5.24*
*Syringic acid*	*2.18*	*Gallic acid*	*6.72*
*Rutin*	*4.14*	*Caffeic acid*	*8.26*
			
			
*Kaempferol*	*2.47*	*Quercetin*	*1.67*
*Quercetin*	*1.18*	*Methyl gallate*	*6.26*
		*Rutin*	*5.48*

**Table 4:** Interaction of the phytochemicals selected from the docking analysis with the target proteins.

Compounds	Protein Targets	Hydrogen Bonds	Hydrophobic Interaction
		Interacting Residues	Interacting Residues
*Donepezil*	*4EY7*	*Phe295*	*Trp86 Phe338 His447 Tyr337 Tyr341 Trp286*
*Myricetin*		*Tyr133 Ser203 Glu202 Gly126 Trp86 Ser125 Gln71 Tyr72 Asp74 Tyr341 Tyr337*	*Trp86 Tyr124*
*Quercetin*		*Glu202 His447 Tyr341 Tyr337 Ser125 Asp74 Tyr72 Gln71 Trp86 Gly126 Tyr133*	*Tyr124 Trp86*
*Decamethonium*	*6EP4*		*His438 Trp82 Asp70 yr332 Thr120 Asn83*
*Rutin*		*Trp82 Tyr128 Thr122 Glu197 Ser198 Ser287 Thr120 Gly116 Gln119 Ala328 Asn68 His438 Asn83 Trp430 Gly78 Asp70*	*Phe329 Trp430 Ala328*
*Quercetin*		*Tyr440 Trp430 Gly78 Trp82 His438 Tyr128 Glu197 Thr120 Gln119*	*Trp82 Gly115*
*Safinamide*	*2V5Z*	*Gln206*	*Cys172 Ile316 Ile199 Tyr326 Tyr398 Leu171*
*Kaempferol*		*Pro120 Ile199 Ty326 Gln206 Cys172 Ile198 Tyr4335 Leu164*	*Ile316 Leu171 Tyr326 Ile198 Ile199 Cys172*
*Quercetin*		*Tyr435 Cys172 Pro102 Tyr326*	*Ile199 Leu171 Cys172 Tyr326 Tyry398*

**Table 5:** The means and standard deviations of several metrics that were examined from the MDS trajectories of the top-docked in complex with their corresponding targets.

*Complexes*	*Thermodynamic Parameters*				
	*RMSD (Å)*	*RMSF (Å)*	*SASA (Å^2^)*	*RoG (Å)*	*H-Bonds*
*4EY7_Donepezil*	*1.68 ± 0.24*	*0.87 ± 0.71*	*23,047.9 ± 465.99*	*23.20 ± 0.10*	*114.39 ± 9.33*
*4EY7_Quercetin*	*1.71 ± 0.21*	*0.85± 0.41*	*23,048.4 ± 49.53*	*23.19 ± 0.09*	*114.90 ± 8.41*
*4EY7_Myricetin*	*1.72 ± 0.22*	*0.84 ± 0.53*	*23,147.3 ± 487.87*	*23.22 ± 0.09*	*114.53 ± 9.41*

**Table 6:** The means and standard deviations of several energy components that contribute to the free energy of binding between top-docked phytochemicals and target proteins.

SYSTEM	Δ_VDWAALS_	Δ_EEL_	Δ_EGB_	Δ_ESURF_	Δ_GGAS_	Δ_GSOLV_	Δ_TOTAL_
*4EY7_Donepezil*	*−42.77 ± 3.09*	*−9.72 ± 11.54*	*38.57 ± 10.55*	*–5.99 ± 0.417*	*−52.59 ± 12.34*	*32.7 ± 11.5*	*−19.92 ± 3.62*
*4EY7_* *quercetin*	*−31.26 ± 4.20*	*−15.10 ± 7.52*	*28.27 ± 5.27*	*−4.10 ± 0.54*	*−46.38 ± 9.35*	*24.20 ± 5.10*	*−22.20 ± 5.19*
*4EY7_* *Myricetin*	*−38.23 ± 3.74*	*−34.178± 10.25*	*51.67 ± 6.76*	*−5.10 ± 0.31*	*−72.37 ± 9.56*	*46.59 ± 6.75*	*−25.78 ± 4.04*

## Data Availability

The datasets generated during and/or analyzed during the current study are available from the corresponding author upon reasonable request.

## Supplementary Materials

The following supporting information can be downloaded at: https://www.mdpi.com/article/10.3390/life14101222/s1, Figure S1: Chromatogram of crude formulation A; Figure S2: Chromatogram of crude formulation B.
