# Supplementary material for: Network Pharmacology, Molecular Dynamics and In Vitro Assessments of Indigenous Herbal Formulations for Alzheimer’s Therapy
Source: Life (Basel). 2024 Sep 25;14(10):1222. doi: 10.3390/life14101222 (PMC11508826; doi:10.3390/life14101222)
Supplement: Supplementary file 1 [file life-14-01222-s001.zip › life-3183682-supplementary.pdf]

Network Pharmacology, Molecular Dynamics and In Vitro Assessments of Indigenous Herbal Formulations for Alzheimer's Therapy

Oluwafemi Adeleke Ojo [1,2,*], Omolola Adenike Ajayi-Odoko [3], Gideon Ampoma Gyebi [4,5], Damilare IyinKristi Ayokunle [6], Akingbolabo Daniel Ogunlakin [1,2], Emmanuel Henry Ezenabor [1,2], Adesoji Alani Olanrewaju [7], Oluwatobi Deborah Agbeye [1,2], Emmanuel Tope Ogunwale [8], Damilare Emmanuel Rotimi [9,10], Dalia Fouad [11], Gaber El-Saber Batiha [12] and Oluyomi Stephen Adeyemi [1,2,13]

1 Good Health and Wellbeing Research Clusters (SDG 03) Bowen University, Iwo 232102, Nigeria; gbolaogunlakin@gmail.com (A.D.O.); emmanuel.ezenabor@bowen.edu.ng (E.H.E.); oluwatobiagbeyed@gmail.com (O.D.A.); oluyomiadeyemi@gmail.com (O.S.A.)
2 Phytomedicine, Molecular Toxicology, and Computational Biochemistry Research Group, Biochemistry programme, Bowen University, Iwo 232102, Nigeria.
3 Microbiology programme, Bowen University, Iwo 232102, Nigeria; omolola.ajayi@bowen.edu.ng
4 Natural Products and Structural (Bio-Chem)-Informatics Research Laboratory (NpsBC-RI), Department of Biochemistry, Bingham University, Karu 961105, Nigeria; gideonagyebi@gmail.com
5 Department of Biotechnology and Food Science, Faculty of Applied Sciences, Durban University of Technology, P.O. Box 1334, Durban 4000, South Africa
6 Pure and Applied Biology programme, Bowen University, Iwo 232102, Nigeria; opeoluwa02@gmail.com
7 Chemistry and Industrial Chemistry programme, Bowen University, Iwo 232102, Nigeria; adesoji.olanrewaju@bowen.edu.ng
8 Animal Science programme, Bowen University, Iwo 232102, Nigeria; tope.ogunwale@bowen.edu.ng
9 Department of Pharmacology and Pharmaceutical Sciences, Alfred E. Mann School of Pharmacy and Pharmaceutical Sciences, University of Southern California, Los Angeles, CA, United States of America.
10 Department of Biochemistry, Landmark University, Omu-Aran 251101, Nigeria; rotimidamilare1@gmail.com
11 Department of Zoology, College of Science, King Saud University, PO Box 22452, Riyadh 11495, Saudi Arabia; dibrahim@ksu.edu.sa
12 Department of Pharmacology and Therapeutic, Faculty of Veterinary Medicine, Damanhour University, Damanhour 22511, AlBeheira, Egypt; gaberbatiha@gmail.com
13 Laboratory of Sustainable Animal Environment Systems, Graduate School of Agricultural Sciences, Tohoku University, Sendai 980-8579, Japan

*Corresponding authors: OAO: oluwafemiadeleke08@gmail.com

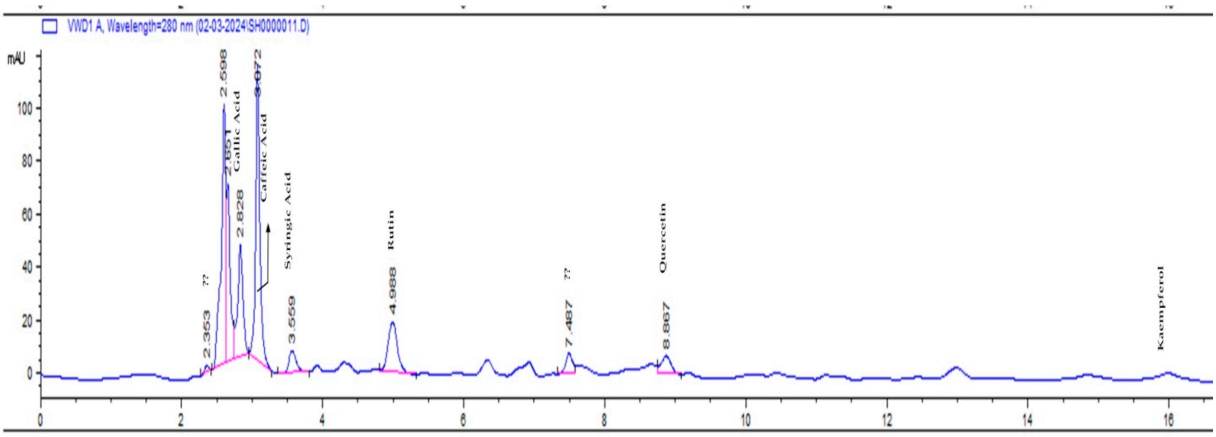

Figure S1: Chromatogram of crude formulation A

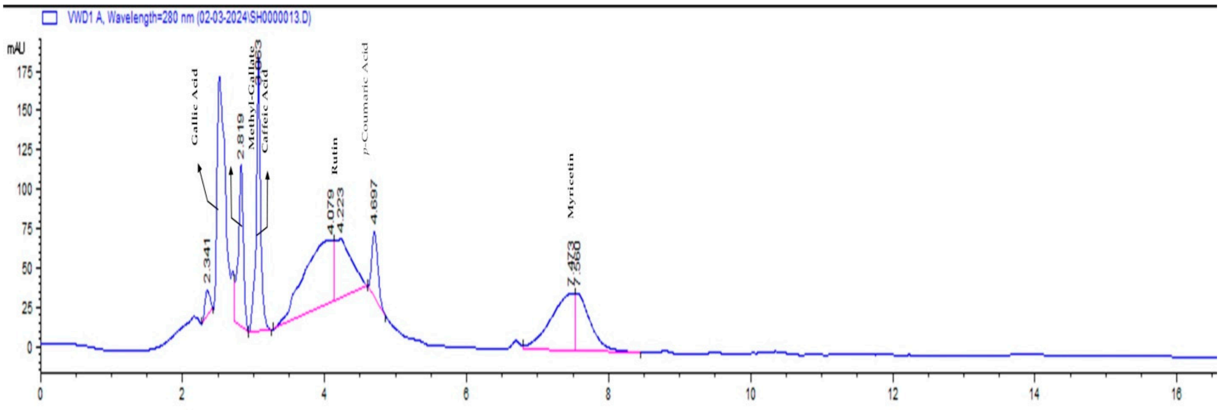

Figure S2: Chromatogram of crude formulation B
